# Workflow-driven clinical decision support for personalized oncology

**DOI:** 10.1186/s12911-016-0314-3

**Published:** 2016-07-21

**Authors:** Anca Bucur, Jasper van Leeuwen, Nikolaos Christodoulou, Kamana Sigdel, Katerina Argyri, Lefteris Koumakis, Norbert Graf, Georgios Stamatakos

**Affiliations:** 1 Precision and Decentralized Diagnostics, Philips Research, Eindhoven, The Netherlands; 2 National Technical University of Athens, ICCS, Athens, Greece; 3 Computational BioMedicine Laboratory, FORTH-ICS, Heraklion, Greece; 4 Department of Pediatric Oncology and Hematology, Saarland University, Homburg, Germany

**Keywords:** Clinical decision support, Clinical workflows, Knowledge models, CDS adoption, Oncology

## Abstract

**Background:**

The adoption in oncology of Clinical Decision Support (CDS) may help clinical users to efficiently deal with the high complexity of the domain, lead to improved patient outcomes, and reduce the current knowledge gap between clinical research and practice. While significant effort has been invested in the implementation of CDS, the uptake in the clinic has been limited. The barriers to adoption have been extensively discussed in the literature. In oncology, current CDS solutions are not able to support the complex decisions required for stratification and personalized treatment of patients and to keep up with the high rate of change in therapeutic options and knowledge.

**Results:**

To address these challenges, we propose a framework enabling efficient implementation of meaningful CDS that incorporates a large variety of clinical knowledge models to bring to the clinic comprehensive solutions leveraging the latest domain knowledge. We use both literature-based models and models built within the p-medicine project using the rich datasets from clinical trials and care provided by the clinical partners. The framework is open to the biomedical community, enabling reuse of deployed models by third-party CDS implementations and supporting collaboration among modelers, CDS implementers, biomedical researchers and clinicians. To increase adoption and cope with the complexity of patient management in oncology, we also support and leverage the clinical processes adhered to by healthcare organizations. We design an architecture that extends the CDS framework with workflow functionality. The clinical models are embedded in the workflow models and executed at the right time, when and where the recommendations are needed in the clinical process.

**Conclusions:**

In this paper we present our CDS framework developed in p-medicine and the CDS implementation leveraging the framework. To support complex decisions, the framework relies on clinical models that encapsulate relevant clinical knowledge. Next to assisting the decisions, this solution supports by default (through modeling and implementation of workflows) the decision processes as well and exploits the knowledge embedded in those processes.

## Background

The large investments in the healthcare industry in improving the capabilities for data capture did not lead so far to solutions able to efficiently deliver to the clinical users knowledge and insight, not only disparate pieces of information [1]. The substantial efforts dedicated to the development of clinical models for personalization in prevention, diagnosis and treatment are still to be leveraged in an integrated, wide-scale approach in clinical care. Additionally, coherent steps towards semantic integration of data [2], and the implementation of high quality and comprehensive Clinical Decision Support (CDS) systems [3, 4], are central to achieving significant improvements in the quality of care, while also reducing the expanding costs.

Meaningful CDS is an especially important prerequisite for reducing the knowledge gap between clinical research and practice in a complex genetic disease such as cancer. Cancers are among the leading causes of morbidity and mortality worldwide [5]. The oncology domain faces the need for complex stratification and patient management, for a multidisciplinary approach with coordination across clinical specialties, and for access to large volumes of data, information and knowledge. Current CDS solutions are unable to support all the complex decisions required for personalized management of cancer patients [6]. Many of the available applications are ad-hoc, single-point solutions. They become quickly obsolete and unmaintainable due to the high rate of change in therapeutic options and knowledge, and are hampered in their adoption by the inability to meaningfully leverage the wealth of data collected for each patient, and all the relevant clinical knowledge. Adoption is limited by difficulties to integrate these CDS systems in the healthcare environments in a non-disruptive manner.

Driven by the need to help bridge the current knowledge gap between research and practice, p-medicine [7, 8] created an infrastructure supporting the transition from current practice towards personalized medicine in oncology. The CDS framework leverages the large datasets available in the project and the modeling efforts with the goal to efficiently bring the new knowledge to the bedside. This framework and its underlying solution for model storage, management and execution is as well a platform for (continuous) validation of existing models on new data.

The requirement to also provide support for clinical processes and not only for clinical decisions has been discussed in literature. Next to reaching the correct diagnosis, the diagnostic process is considered equally challenging [9]. To support the clinical processes and to provide decision support without disrupting the current way of working, we extend our framework with workflow functionality.

In this paper we first describe our CDS framework implemented in p-medicine and the approach to integration of knowledge models to support future-proof CDS, i.e. systems able to grow and adapt with the rapid growth and change of knowledge in the field. Next, we introduce our architecture for workflow-driven CDS extending the current solution, and describe the benefits of providing clinical workflow support to deliver recommendations when and where needed along the continuum of care. The new system integrates a workflow suite and functionality for the storage, management and execution of clinical workflows and for the storage of traces of execution. The knowledge models are integrated and run from the workflow to support decisions at the right point in the clinical process.

In Section II we discuss the role of a CDS system and the key challenges to CDS implementation and adoption as reflected in the literature, and introduce our relevant previous work. In Section III we describe the CDS framework implemented in p-medicine and its key benefits. In Section IV we present our CDS application implementation that uses the framework, and its evaluation with clinical models in two cancer types. In Section V we focus on the need to model and provide support for clinical processes, showing both the intrinsic benefits and the advantages for CDS. In Section VI we summarize the key conclusions.

The role of a CDS system is to assist clinicians in making clinical decisions relevant for a patient case. Significant effort went to the implementation of tools for CDS in the last few decades [9], with many solutions available today. However, their uptake in the clinic has been limited [6]. Current CDS solutions are faced with significant challenges that hamper their adoption. Improving the human-computer interface, prioritizing and filtering recommendations to the user, creating an architecture for sharing executable CDS modules and services, combining recommendations for patients with co-morbidities, and prioritizing CDS content development and implementation are main aspects that need to be addressed [3].

Even when successful in the clinic, CDS solutions were very limited in scope focusing on very specific decisions and implementing a specific model in a particular context. With the evolving understanding in the clinical community, the evidence-based guidelines and protocols are constantly evolving and expanding. Medical professionals often find challenging to find, study and interpret the new scientific discoveries that may impact care. In this context, keeping the CDS tools and their recommendations up to date is a major task. In addition to the maintenance of external knowledge and access to the latest evidence, the ability to efficiently update the CDS tools to take this new evidence into account and apply it seamlessly in the clinical context has a strong impact on adoption. Finally, fast and minimal-cost validation of the CDS tools is essential.

Another key requirement for CDS implementations, typically unfulfilled, is to present the recommendations in a way that “supports and does not interrupt the clinical workflow” [3].

In [10] we introduce our approach to CDS. We report on the implementation in the p-medicine project of the CDS framework and of the underlying solution for storage, management and execution of knowledge models. The solution relies on clinical knowledge encapsulated in models and has in our view many advantages compared to building monolithic and closed CDS solutions: It is modular, scalable, can be efficiently customized and updated, and can benefit of the modeling efforts of a large community. In this implementation we consider two important sources of models: (i) Validated knowledge described in the literature and (ii) models derived on the comprehensive datasets from clinical trials and care available through the p-medicine infrastructure (which can be used in care after prospective validation). In [11] we reported on the development and integration into the CDS framework of predictive models developed by mining a clinical trial dataset available in p-medicine. We demonstrated that the framework enables efficient collaboration among clinical researchers, knowledge modelers, data miners and CDS implementers. An example of an implemented literature-based model is the St. Gallen stratification for early breast cancer [12].

## Methods

We propose a CDS framework that can effectively address several challenges related to the implementation and adoption of decision support in healthcare. Our objectives are to bring to clinical care the knowledge models developed in the p-medicine project, to enable the reuse of existing models, and to keep up with new clinical knowledge and with the development of new models, leveraging collaboration among modelers, CDS implementers, biomedical researchers and clinicians. In this section we describe our solution and the key benefits of this approach.

### Delivering meaningful and effective CDS

With the continuous growth of medical knowledge and with the introduction of new personalized treatments the CDS recommendations will unavoidably need to evolve and change. The modular approach (knowledge is encapsulated in models with specified input/output instead of monolithic implementation) allows the system to efficiently keep up with introduction of new clinical knowledge in the domain. It also allows easy reuse of models in different clinical contexts (e.g. models relevant for different diseases).

The solution may improve quality of care by the inclusion of advanced clinical models to support decisions. Through access to patient data, outcomes can be monitored and analyzed leading to continuous validation of the models in clinical care. The modular, light-weight way of integrating new models into the CDS applications lowers the threshold for adoption, and the effort of implementation and maintenance.

### The implementation of the CDS framework

**Fig. 1:**
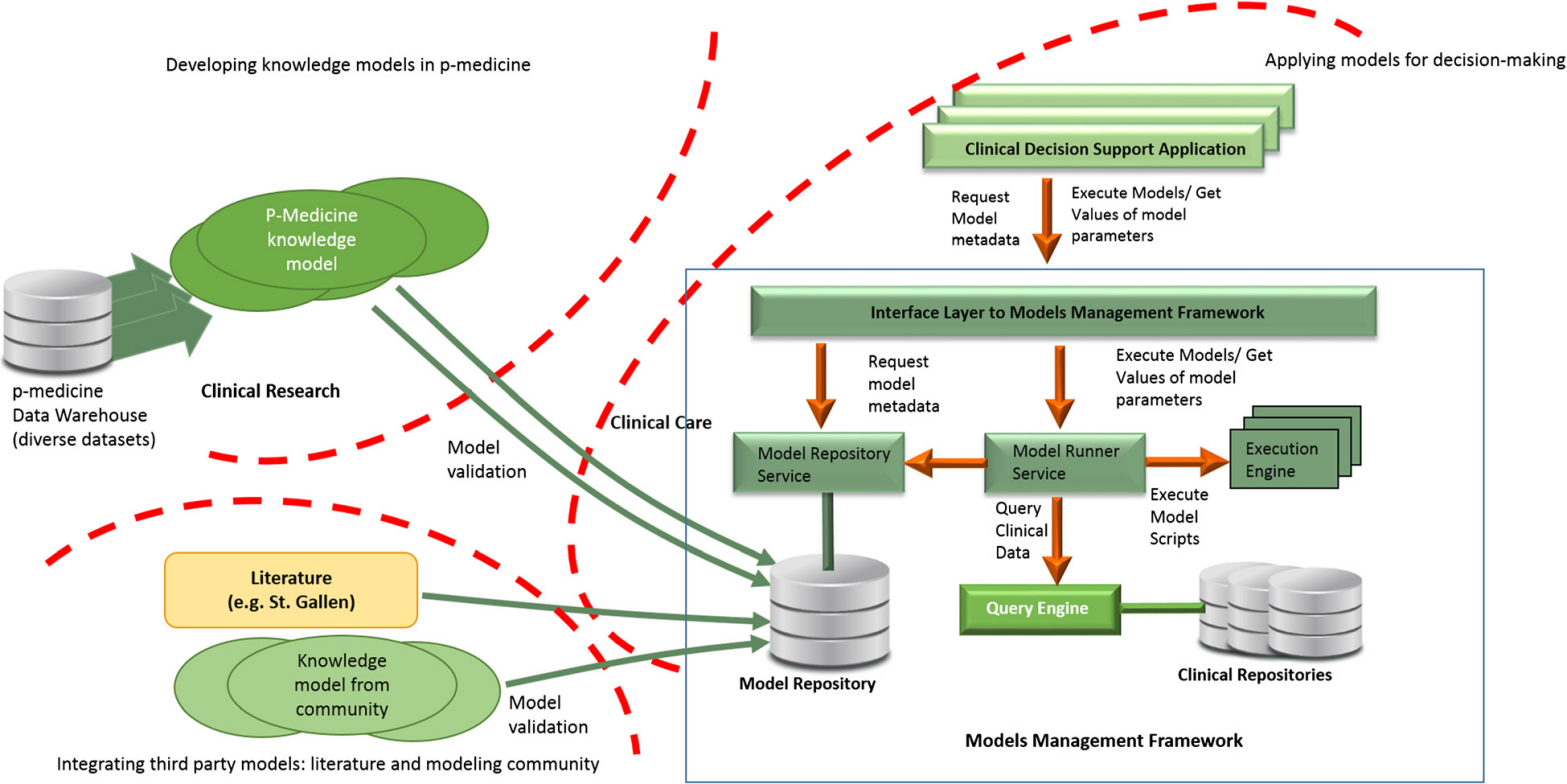
Architecture and context of use of the CDS framework

Figure 1 depicts the overall (informal) architecture of the CDS framework implemented in p-medicine. Next to depicting the main component blocks of the framework, we describe the environment and the context of use of the system. The p-medicine project covers both clinical research and clinical care contexts. The p-medicine data warehouse aggregates the large oncology datasets available in the project. The data are used to develop models that range from predictive models derived by data mining techniques [11] to complex multiscale VPH models [13]. To support the complex decisions required for treating cancer patients no single model is sufficient. A CDS solution needs to integrate all models relevant for decisions in a disease and use them to provide recommendations at the point of care. Therefore, we need to leverage as well knowledge generated outside the project. To this end, we provide mechanisms for easy integration of third party models. We implement literature-based models that are relevant for our scenarios and collaborate with the modeling community to integrate validated models developed outside the project.

While the development of the models is carried out in the clinical research domain, the CDS framework needs to support clinical care. The role of a CDS application is to apply clinical models to support decisions in clinical practice. In this context, validation of the models is an essential prerequisite to their use in a clinical care context.

Models uploaded to the CDS framework are exposed through a uniform interface to be queried by CDS applications. While we developed a CDS application in p-medicine, by loosely coupling the CDS applications and the model management functionality, and by providing a uniform interface to retrieve and execute models we facilitate the use of the underlying framework by third-party CDS implementers. This also opens the framework to customizations of the visualization component to suit the needs of each clinical site.

The solution facilitates continuous validation and update of existing models on new datasets when those become available in the p-medicine infrastructure. The validation of individual models included in the CDS framework can be carried out without disrupting the operation of the system.

The Model Repository contains descriptions of models (e.g. purpose, parameters), their specification (e.g. executable script) and all the relevant metadata (e.g. version, authors). The models can be either fully specified by storing in the Model Repository code that can be executed by one of the generic engines deployed in the framework (e.g. Groovy [14] scripts), or specified by a reference to a dedicated service implementing the model. New engines can be deployed in the framework when needed.

The Model Repository Service exposes the models metadata through the Interface Layer, and allows the Model Runner Service to access the model specifications. The model execution is initiated from the CDS application. When models are executed, the Model Runner Service gets the patient data from the clinical repositories (e.g. Electronic Health Record System) or from user input through the CDS application, and the model specification from the Model Repository Service. Next, the Model Runner Service initiates the execution of the model on the suitable generic Execution Engine or by the dedicated service. The results of the model (model output) are delivered to the CDS application and displayed (e.g. as a recommendation). The CDS framework currently integrates models in the oncology domains covered by the p-medicine scenarios [8].

## Results and discussion

### The CDS application implementation

Leveraging the underlying CDS framework we implemented a CDS application to address the needs of the p-medicine clinical users.

#### The front-end application

The application navigates the user through the phases of patient management, retrieves and displays the patient data, enables users to inspect, customize and run the deployed models, and provides model descriptions and links to relevant external information (e.g. literature, guidelines).

**Fig. 2:**
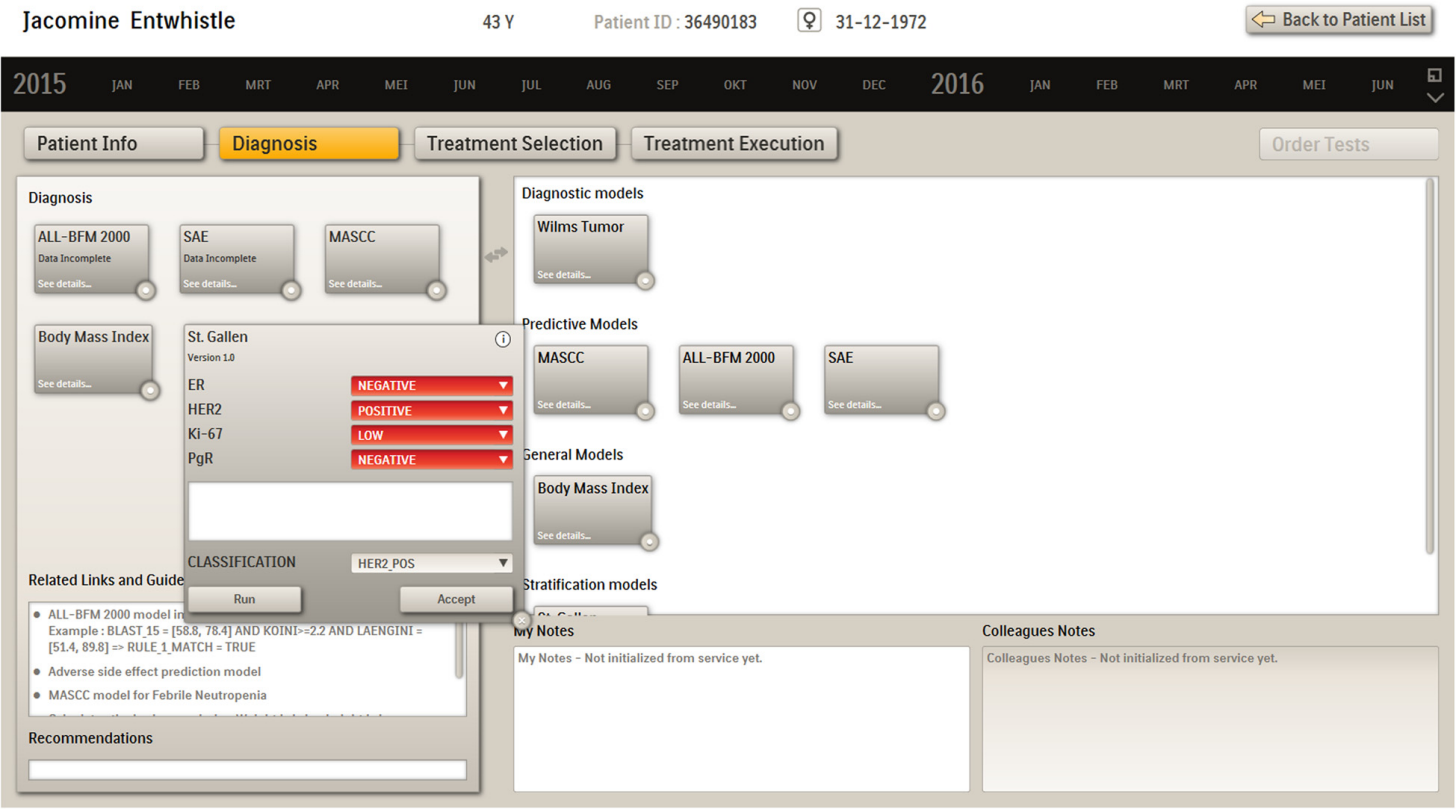
Diagnosis screen

As seen in Fig. 2, the application includes first steps towards supporting clinical processes by distinguishing several phases of patient management: data review, diagnosis, treatment selection; models are incorporated in the relevant stage and the information flow between stages is supported.

Models are selected by moving them from the right panel to the left panel to be instantiated with the relevant patient data (from the patient file or user input), and applied for the current patient. The clinician can accept or override the model recommendation. The recommendation appears in the corresponding box and can be extended by the clinician and saved to the patient file. Recommendations and decisions are persisted by the tool for evaluation (of both tool and models), analysis, and quality monitoring.

**Fig. 3:**
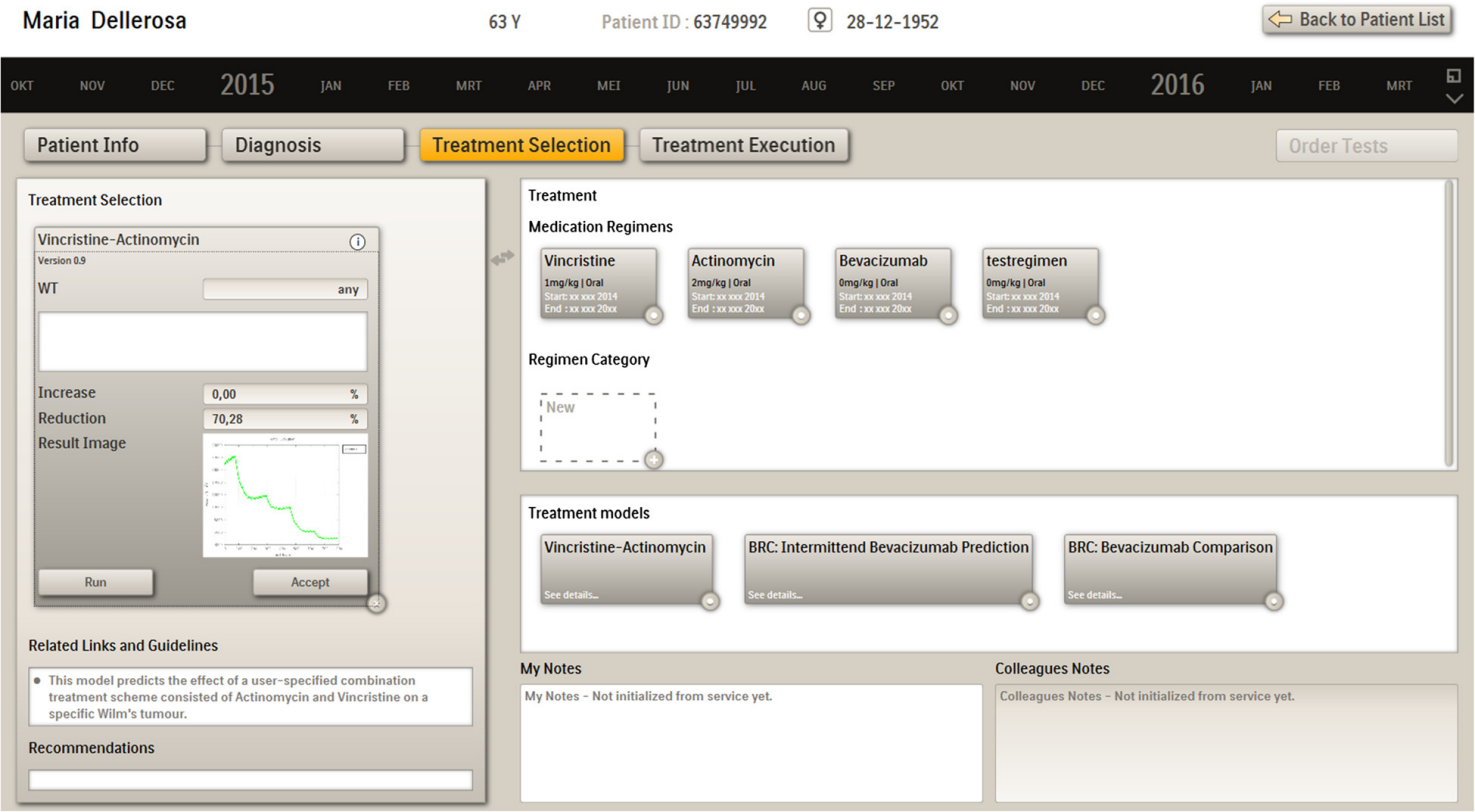
Treatment selection screen

Figure 3 shows the treatment selection screen including a range of treatment models and medication templates. The clinician selects the suitable treatment models. Based on the results of the models applied in diagnosis phase the system may suggest specific treatment models as applicable. Recommendations of the treatment models may refer to the most effective treatment for this patient, safety risks, comparisons of different treatments, etc. The medication templates can be modified, further specified e.g. with specific substances or detailed doses and ways of administration, and saved for future reuse. They are moved to the left panel to be instantiated with patient data and prescribed.

#### Evaluation scenarios

We evaluated the CDS (front-end) application implementation and the underlying framework in scenarios in two diseases of focus in p-medicine, breast cancer and nephroblastoma. In each scenario, relevant knowledge models (developed in p-medicine or selected from literature) addressing diagnosis and treatment selection were deployed in the CDS framework and integrated in the CDS application.

We previously demonstrated the ease of deployment of new models into the framework [15]. In this evaluation the focus was on evaluating the efficiency of building a CDS application providing meaningful support that uses the framework and incorporates deployed models. The scenarios were defined with the clinical users in the project and the CDS application was well received by the clinicians in the two clinical domains. This evaluation has shown the feasibility of the overall approach as well: Both model integration in the CDS application and model deployment in the framework require relatively low implementation effort. Models can be easily replaced and updated, reused, and combined.

#### p-medicine knowledge models

In this section we describe several p-medicine models that have been deployed in the CDS framework and used to evaluate the CDS application implementation and the framework.

In future work, the models will be prospectively validated with new data in the context of clinical studies. The CDS application and the underlying framework will support the efficient execution of the models with the new patient data and preserve the results. Next to validating the model, this allows to demonstrate the ability of the framework to support the efficient (continuous) validation of the models.

#### The Oncosimlator models

The objective of the Oncosimulator is to simulate the response of clinical tumours to specific treatment schemes and/or schedules in patient individualized context [13]. In the CDS system, the Oncosimulator is represented by two branches addressing breast cancer and nephroblastoma.

The breast cancer branch deals with the paradigm of anti-angiogenic treatment and specifically of single-agent bevacizumab therapy. A continuum approach describing vascular tumour growth under angiogenic signaling has been developed based on relevant literature [16] and bevacizumab pharmacokinetic properties have been incorporated [17]. In particular, the model consists of a pair of ordinary differential equations reflecting the interplay between tumour volume and carrying capacity i.e. the maximal tumour volume that the current vascular system can support. The nephroblastoma Oncosimulator model [18, 19] is a predominantly discrete, clinically-oriented multiscale cancer model simulating the tumor response to preoperative chemotherapy schemes based on the SIOP clinical trial. Different scenarios have been formulated addressing specific clinical questions. From these scenarios, three have been selected for CDS integration, two utilizing the breast cancer model and one utilizing the nephroblastoma model.

**Fig. 4:**
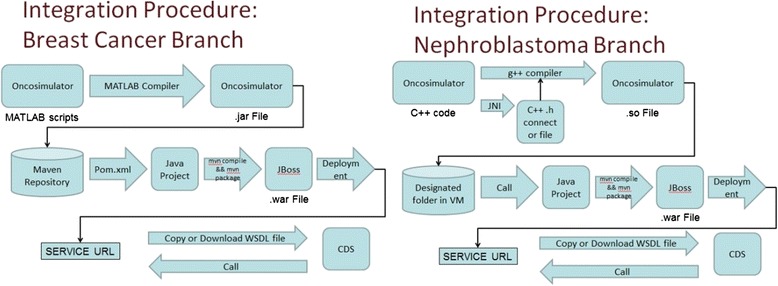
CDS integration of breast and nephroblastoma branches

The integration of the Oncosimulator models is based on publishing models as dedicated engines. As the two branches were implemented in different programming languages, the models were incorporated in a wrapper to achieve the required integration and build the services. For each Oncosimulator branch a different procedure of transformation and integration was followed (Fig. 4). The breast cancer branch is developed in MATLAB, as a master script, calling a number of functions. Therefore, by utilizing the MATLAB Compiler, the model was recompiled into an executable java jar, with its main runner class being mapped to the master script. The jar was finally included in the wrapper’s Maven repository. The nephroblastoma branch, written in C ++, was recompiled in a form of shared library using the Java Native Interface, by changing its main function into a function which communicates with the wrapper code through a header file. Both procedures produce corresponding wsdl files, which describe the web services.

**Fig. 5:**
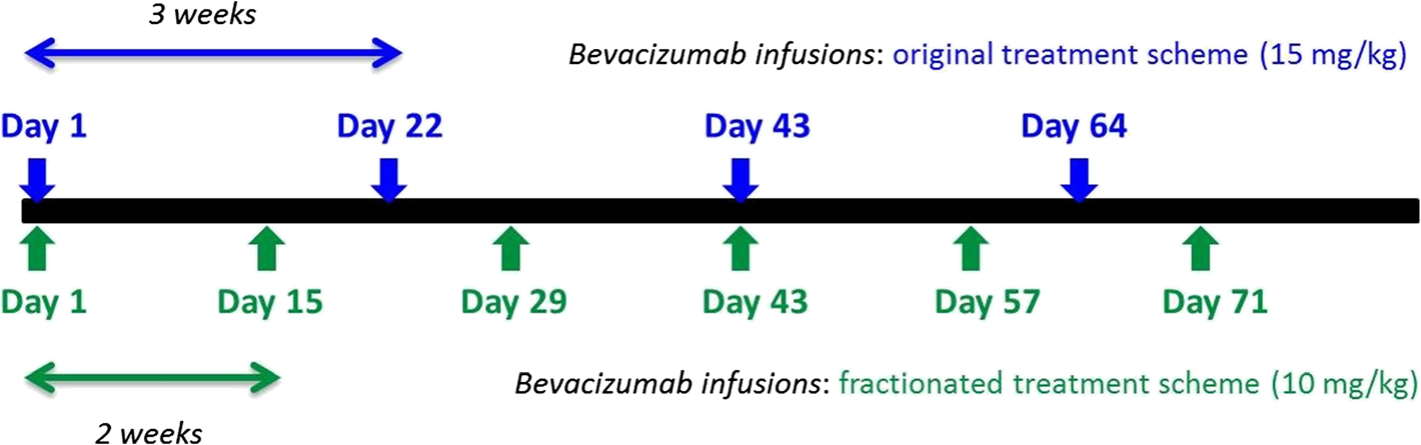
Details of two simulated bevacizumab monotherapy schemes (dosage, frequency of administration and timepoints of administration). The treatment scheme details are extracted from European medicines agency (Avastin, INN - bevacizumab - WC500029271.pdf) and constitute the two suggested modes of bevacizumab administration for metastatic breast cancer

**Fig. 6:**
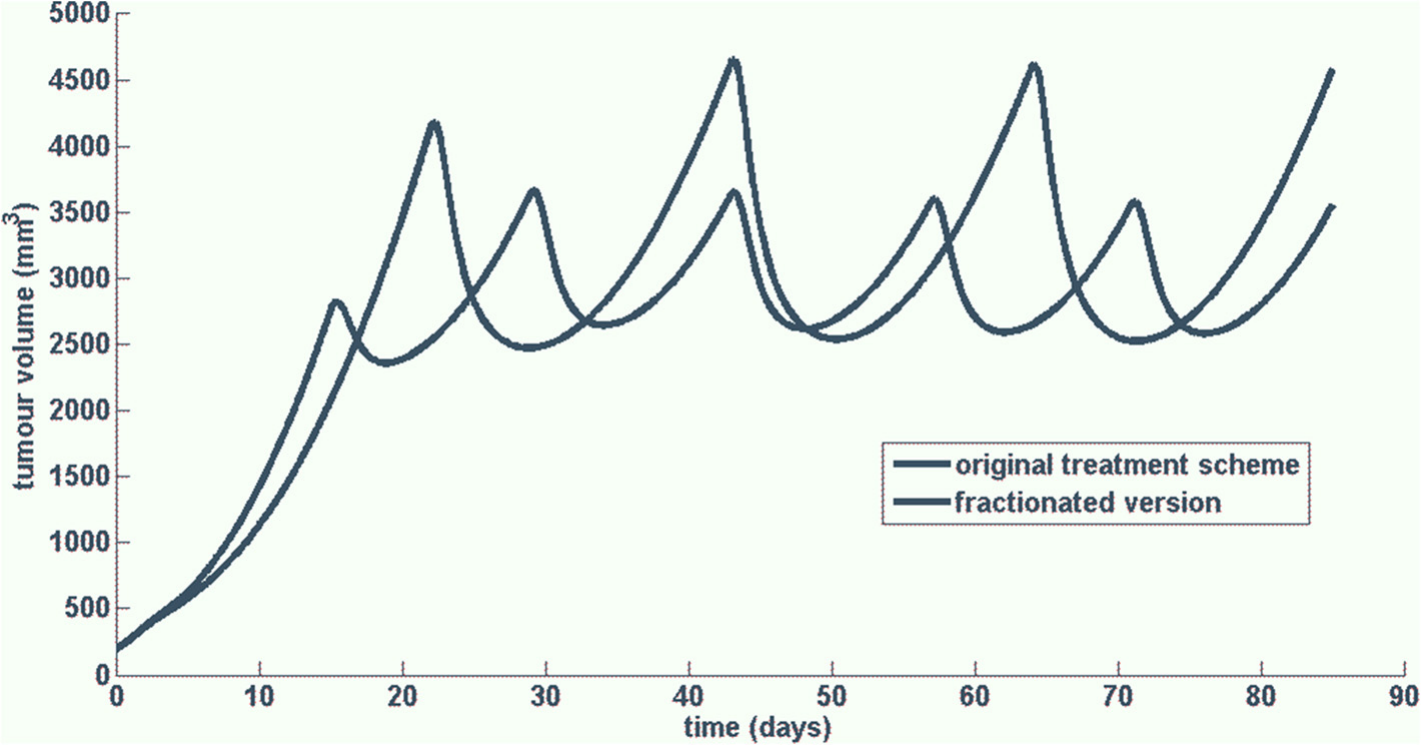
Tumour volume time evolution for a tumour treated with bevacizumab according to a treatment scheme consisting of the administration of 15 mg/kg of bevacizumab as a single-agent, once every three weeks for a total of 4 doses (blue line) and a fractionated version of the aforementioned treatment scheme consisting of the administration of 10 mg/kg of bevacizumab as a single-agent, every other week for a total of 6 doses (green line)

To demonstrate the integration of the Oncosimulator into the CDS system, an indicative CDS scenario on breast cancer, addressing an actual clinical question regarding the impact of applying fractionated versions of an original treatment scheme on the treatment outcome, is outlined below. The clinician selects the corresponding model (breast cancer scenario) and sets values to user generated input parameters (treatment schema) (Fig. 5). By pressing run, a request is called to the remote service. The request is sent to the URL of the remote machine where the model was published, together with the necessary input data. Based on the input data, a file is created (csv for breast cancer, xml for nephroblastoma), which serves as input to the model. After the execution is completed, the output files are saved into the remote machine and a predefined set of results depicting clinically important information (the required set of biological values, along with a graph depicting the tumor evolution over time), are sent as response and shown in the CDS’s GUI (Fig. 6).

The scenario is presented via an exemplar case study based on a model instance already developed using time-course data derived in the context of in vivo experiments studying the anti-tumour efficacy of bevacizumab in higher mammalian species (Mus musculus). The way that the clinician interacts with the system is sufficiently generic and as such will not be affected by the substitution of this model instance with another referring to human patient cases and which is currently under refinement. The details of both the original and the fractionated version of the scheme appear in Fig. 5.

Even though the treatment schemes did not induce tumour regression for the specific tumour, tumour growth inhibition (i.e. comparing to the tumour evolution in the case of untreated growth) has been achieved for both cases. In particular, as it is shown in Fig. 6, the original treatment scheme (blue line) has induced a tumour growth inhibition percentage equal to 73.6 % while the fractionated one (green line) has prompted a tumour growth inhibition percentage equal to 79.5 % (calculated as the percentage of change in tumour volume in the treatment module simulation with respect to the free growth module simulation, at the end of the treatment cycle). Hence, the superiority of the fractionated scheme comparing to the original scheme would be revealed to the user along with the implied statement that none of the applied modes of administration would induce tumour regression.

#### miRNA model for nephroblastoma

MicroRNAs (miRNA) have been implicated in a number of diseases, including cancer, and dysregulation of them has been widely observed in different stages of cancer [20, 21]. The regulation of miRNAs can result in post-transcriptional down-regulation or up-regulation of the expression of certain genes [22], which subsequently affect other genes downstream in gene regulatory networks (GRNs) or biological processes. Taking into account miRNA expression data from a patient and GRNs knowledge, the treating physician may select targeted drugs for personalized treatment to improve efficiency and increase quality of care.

The miRNA pathway analysis model was incorporated in the CDS framework to support the evaluation in the nephroblastoma scenario. During diagnosis, the miRNA model supports the non-intrusive early identification of nephroblastoma patients among the tested subjects. The model was created using a public miRNA dataset including 23 nephroblastoma patient samples (prior to chemotherapy) and 19 healthy controls [23] (GSE38419) in conjunction with the human pathways from the KEGG database [24]. The model identifies the targeted/disrupted genes from the miRNA expression data and using the MinePath pathway analysis system [25] extracts the functional and nonfunctional sub-paths of the healthy and diseased samples. Finally, a predictive model using the C4.5 decision tree algorithm [26] on the functional status of the sub-paths is created. More details of the model including setup and algorithmic steps are described in [27]. The assessed performance of the predictive model is 80 % for 10-fold cross-validation, and 78 % for leave one out cross validation.

The decision tree highlighted three key sub-paths including hub genes (highly connected genes) in signaling pathways, able to discriminate between nephroblastoma and healthy subjects. Specifically, in the GnRH signaling pathway the sub-path PLCβ → PKC β → MEKK has been selected as significant for the healthy population, an outcome which is in accordance to the literature since the protein kinase C (PKC) is implicated in the regulation of neuroblastoma [28]. The sub-paths PDK1 β → AKT β → CREB in the PI3K-AKT signaling pathway and the P50 β → COX2 in NF-KAPPA B pathway have been also selected as significant for the model, which are known as regulators for neuroblastoma cell survival [29] and proliferation [30] respectively.

### Workflow-driven CDS

In this Section we introduce our novel approach to CDS provision which supports both the clinical decisions and the clinical processes. The workflow-driven CDS framework builds on the CDS framework described in Section III and extends it with capabilities for workflow modeling, management and execution. For each clinical domain of relevance, the clinical processes for the specialists involved in care provision are modeled and implemented in the framework. Clinical knowledge models are incorporated in the workflows to provide recommendations at the right decision points within the clinical processes.

Healthcare organizations deploy and adhere to well-defined clinical processes developed and optimized over time, derived from evidence, and based on the clinical guidelines and protocols built by the clinical community to ensure that the best care is provided. Studies have shown that in oncology treatment according to an established protocol assures the best available care for a patient [31]. Such protocols have been widely implemented in clinical care and often customized to the local context of each clinical site. We formalize clinical processes into clinical workflows, which can be modeled and optimized. We use open source tools previously developed for business processes such as jBPM [32], leveraging the Business Process Model and Notation (BPMN) standard [33].

In the rest of the section we discuss the relevance of workflow support for CDS, then we briefly describe our workflow-driven CDS framework (detailed in [13]).

#### Relevance of clinical workflows for CDS implementation

Deployed clinical processes include both explicit knowledge (e.g. rules and tasks derived from protocols or specific to the local hospital context), and implicit information captured in their implementation within a healthcare organization. When leveraging the clinical processes a CDS solution benefits of all the knowledge, both explicit and implicit.

Integrating CDS functionality in the established processes can help address the complexity of personalized patient management along the entire care continuum in a flexible and scalable way. This approach enables seamless integration in the clinical environment and cost-effective adaptation and customization. Adherence to the local clinical processes leads to a low adoption barrier for the CDS by avoiding disruption of the way of working. Building decision support that does not interrupt the clinical workflow has been identified as an important factor for the success of a CDS solution [3]. Driving the CDS provision from the clinical workflows also facilitates dissemination of best practices and of the newest protocols and knowledge, as it enables clinical sites to efficiently share workflows, enrich them, and customize them for local needs.

#### Workflow-driven CDS framework

To support efficient decision making in complex clinical scenarios, such as those driving care delivery in oncology that involve several specialists and a large number of interrelated and coordinated steps, we model and implement the clinical processes that underlie each scenario. We build a dynamic system able to provide for each patient the right recommendation at the right time to the right clinical specialist.

We propose a modular approach allowing for efficient extensions to new clinical domains. For each relevant disease we define modules corresponding to coordinated care processes (e.g. protocols) and represent the workflows of all specialists involved (e.g. oncologist, nurse, pathologist) and their interactions. These multispecialty workflow modules are stored in the Workflow Models Repository. We also represent customizations designed by the healthcare organizations and build mechanisms for handling deviations from the defined processes (e.g. to handle cases when the standard protocol is not applicable or an exception has occurred).

Each workflow module has its own specific decision support needs which we support by incorporating suitable knowledge models at the right steps. The framework facilitates multi-site collaboration as well and supports alternative workflows in each module. Deviations from the specified workflows are stored, managed and analysed to help improve the clinical processes. Changes to clinical workflows and to the knowledge models can be efficiently implemented. This solution delivers scalability both with respect to workflows definition and to deployment of knowledge models.

**Fig. 7:**
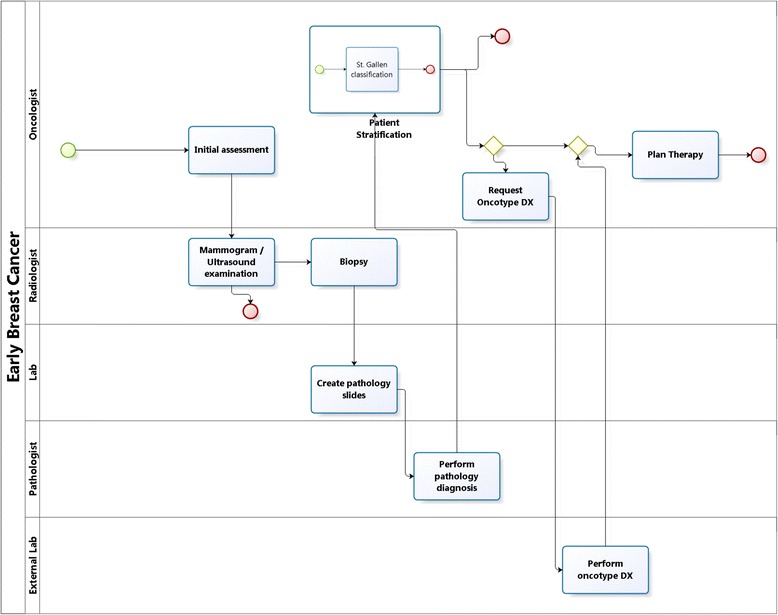
Simplified workflow for early Breast Cancer involving several specialists and departments. The workflow is represented using BPMN [20]

Figure 7 depicts a simplified workflow for the management of early breast cancer according to the St. Gallen guidelines [12]. Several specialists are involved with specific clinical processes: treating physician/oncologist, pathologist, radiologist, laboratory (technician). We represent tasks, decision points, models executed in those decisions points to generate recommendations, and the interactions among the clinical experts. In the example, a patient is assessed by the oncologist and a radiology examination is ordered. If no suspected lesion is identified the workflow stops. Otherwise, pathology diagnosis is ordered. If the diagnosis is negative the process ends. Otherwise, the results are fed into the St. Gallen model to stratify the patient. Applying the St. Gallen guidelines, for several disease subtypes the oncologist orders an additional test (Oncotype DX [34]) performed by a dedicated lab. Combining the results, the oncologist plans the therapy.

**Fig. 8:**
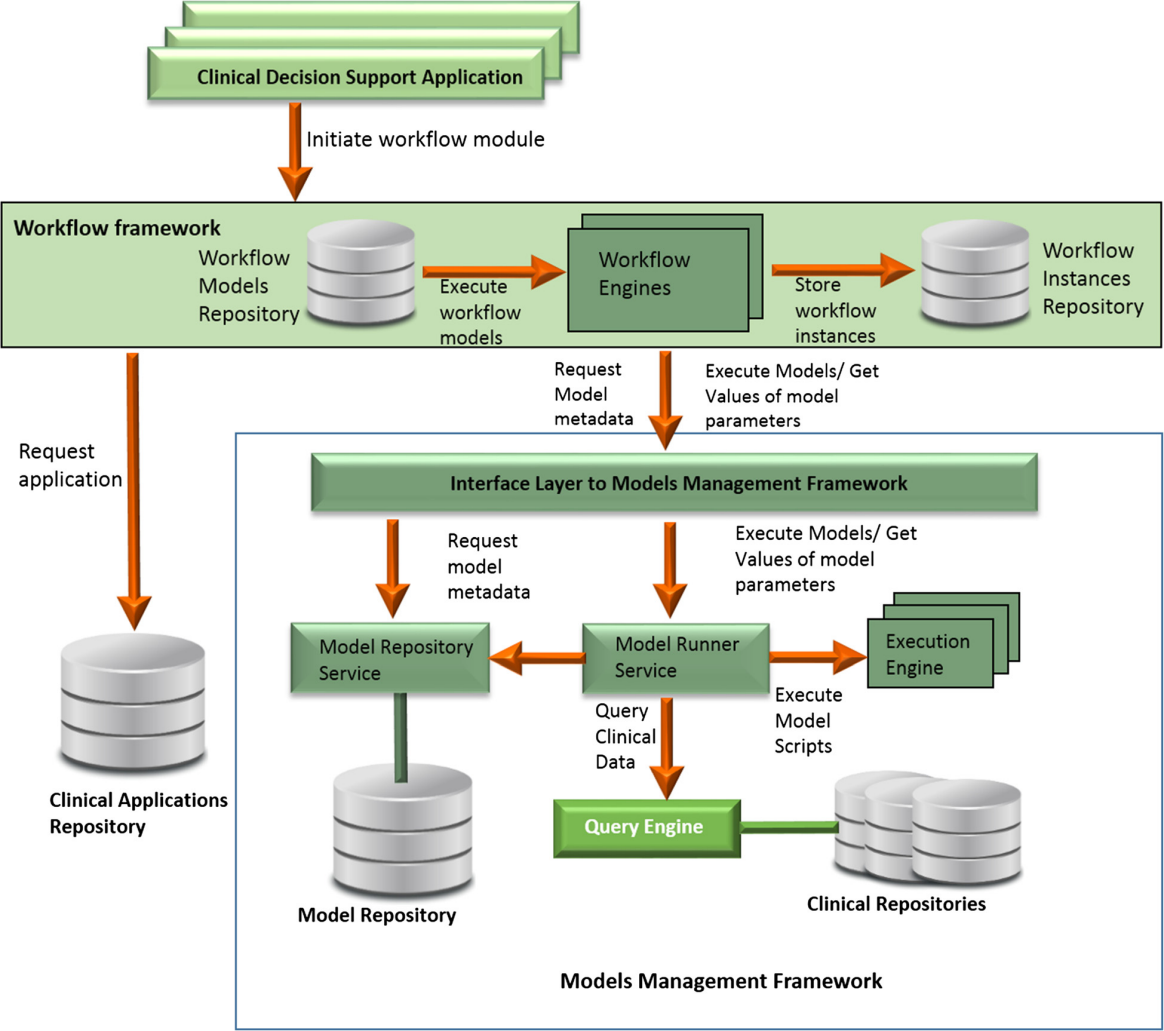
The overall architecture of the workflow-driven CDS framework

Figure 8 depicts the overall architecture of the workflow-driven CDS framework. When providing support for a specific patient case, the suitable workflow module is retrieved from the Workflow Models Repository. The workflow models in this module will be executed by engines deployed in the workflow environment (e.g. locally at the healthcare organization). A set of workflow suites may be evaluated to select the most suitable components (engines, editors, etc.) for each deployment. For the initial implementation we have selected jBPM due to its adoption and open source license.

Executed workflow instances are persisted to be used to evaluate the performance of the system, the quality of the recommendations and the users’ behaviour and interaction with the system. The aggregation and analysis of the clinical process data can provide significant insights that go beyond the clinical processes alone, having as well the potential to improve the outcomes and the quality of care. For workflow execution the framework relies on several services that retrieve and run relevant clinical models as described in Section III.

## Conclusions

In this paper we present our CDS framework developed in p-medicine and the CDS implementation leveraging the framework. To support complex decisions, the framework relies on clinical models that encapsulate relevant clinical knowledge. The p-medicine project builds a range of such models of various complexity and we have also implemented literature-based models.

Our solution was designed to provide flexibility and enable keeping up with the steep growth and change of clinical knowledge in oncology. The CDS application implementation allowed to demonstrate the capabilities of the framework. We evaluated it in two clinical scenarios in breast cancer and nephroblastoma, applying both knowledge models developed in p-medicine and literature-based.

Further, we extended this framework to leverage and support the established clinical processes. Next to the traditional business processes, healthcare environments have their specific clinical processes (often based on clinical protocols that are customized to fit the local context). We model and implement these processes as clinical workflows that drive CDS provisioning. Implementing the clinical processes we lower the adoption barrier of the CDS solution, and exploit the implicit and explicit knowledge contained in these processes to provide better decision support.

This loosely-coupled solution allows to customize for each healthcare organization both the clinical processes and the knowledge models applied for decision support. The approach also enables efficient management of updates to the clinical models to reflect the latest clinical knowledge and to the clinical workflows to apply the most recent clinical guidelines and protocols.

The traces of workflow execution can provide much needed insights concerning existing clinical processes and their implementation. These data can be used to evaluate adherence to established processes and to identify frequent deviations, safety risks, errors, and performance bottlenecks.
